# Exploring the Effects of Problematic Internet Use on Adolescent Sleep: A Systematic Review

**DOI:** 10.3390/ijerph18020760

**Published:** 2021-01-18

**Authors:** Ioulia Kokka, Iraklis Mourikis, Nicolas C. Nicolaides, Christina Darviri, George P. Chrousos, Christina Kanaka-Gantenbein, Flora Bacopoulou

**Affiliations:** 1 Postgraduate Course on the Science of Stress and Health Promotion, School of Medicine, National and Kapodistrian University of Athens, 11527 Athens, Greece; iouliakok@med.uoa.gr (I.K.); cdarviri@med.uoa.gr (C.D.); chrousge@med.uoa.gr (G.P.C.); ckanaka@med.uoa.gr (C.K.-G.); 2 Outpatient Specialty Clinic for Obsessive Compulsive Disorder and Behavioral Therapy, First Department of Psychiatry, Eginition Hospital, School of Medicine, National and Kapodistrian University of Athens, 11528 Athens, Greece; irmour@gmail.com; 3 Division of Endocrinology and Metabolism, Center of Clinical, Experimental Surgery and Translational Research, Biomedical Research Foundation of the Academy of Athens, 11527 Athens, Greece; nnicolaides@bioacademy.gr; 4 First Department of Pediatrics, Aghia Sophia Children’s Hospital, School of Medicine, National and Kapodistrian University of Athens, 15773 Athens, Greece; 5 Division of Endocrinology, Metabolism, and Diabetes, First Department of Pediatrics, Aghia Sophia Children’s Hospital, School of Medicine, National and Kapodistrian University of Athens, 15773 Athens, Greece; 6 Center for Adolescent Medicine and UNESCO Chair on Adolescent Health Care, First Department of Pediatrics, School of Medicine, National and Kapodistrian University of Athens, 15573 Athens, Greece

**Keywords:** internet dependence, sleep deprivation, chronotype deregulation, behavioral addictions, sleep duration, adolescence

## Abstract

Adolescent suse internet via several devices to gather information or communicate. Sleep, as a key factor of adolescents’ development, contributes to their physical and mental health. Over the past decades insufficient sleep among adolescents has been wide spread, and one of its attributing factors is the increased availability of technology. This review aims to investigate the body of evidence regarding the impact of problematic internet use on adolescent sleep. Extensive search of databases was performed according to PRISMA guidelines for studies published within the last decade, regarding subjects aged 10–19. The final step of the search yielded 12 original studies. The quality of extracted data was evaluated with the AXIS tool, in order to estimate the risk of bias. All studies showed a negative correlation between adolescent sleep and problematic internet use. It was found to affect sleep quality and quantity and provoke insomnia symptoms. Interestingly, adolescent’s sex, parental educational level, type of family and use for leisure or academic reasons appeared as affecting factors of the problematic internet use-sleep relationship. Problematic internet use has several effects on adolescents’ sleep. Results of relevant studies should be embedded in educational interventions addressed to adolescents as well as parents, to eliminate the negative outcomes of problematic internet use on sleep and adolescence’s health in general.

## 1. Introduction

There is a wide agreement on the fact that Internet has conquered several aspects of everyday life as it has attained to have an informative, educational and entertaining role for all ages and particularly for adolescents. A cross sectional study among European adolescents showed that 92% of those aged 14 to 17 years have at least one social networking site account and 40% of them spend more than two hours per day connected online [1]. Nowadays, adolescents exhibit a high online engagement and internet is a significant part of their life [2], since they can have access via various devices regardless of time, place and condition. During the past decade, research showed that the prevalence of internet use among adolescence is stunningly high; over 90% of adolescents in the USA and Japan and 72% in China use internet on a daily basis, while internet overuse prevalence has surpassed the 20% for India and Iran among high school students [3]. Problematic internet use (PIU) is defined as “(a) internet maladaptive preoccupation experienced as irresistible use for periods longer than intended, (b) significant distress resulting from behavior and (c) absence of other axis I pathology that might explain the behavior, i.e., mania or hypomania” [4]. Researchers have discovered that internet overuse shares common symptoms (i.e., withdrawal, mood modification and tolerance) with chemical addictions [5] and thereby have outlined the similarities between these behavioral norms [6]. It may intervene in the over-users’ life in various ways, such as influence their interpersonal relationships, cause the loss of internet for other, beneficial activities and even affect essential aspects such as their sleeping routine. A question arises as to where this may lead. Beyond its positive impact, there is scientific consensus around the fact that excessive internet use has negative results, especially during the sensitive developmental period of adolescence [7]. It may stem from a variety of factors, from the emotional, the psychological, or the physiological spectrum. Studies have highlighted that problematic internet use is linked to anxiety, depressive symptomatology and aggressive behavior [8,9,10]. Additionally, prolonged internet use may cause physiological problems such as migraine, headache and back pain [11]. Sleep is considered a crucial component of childhood and adolescent development and health and is dependent on two biological processes. Process C, which refers to the circadian clock, determines when an individual is expected to sleep and wake naturally in a 24 h circle, and Process S constitutes the stimulus for sleep and is determined by the time the individual has been awake [12]. The importance of sleep during adolescence is well documented, as it functions as a regulator for many neurobiological procedures [13]. Sleep has been proven to have a major influence in the metabolic, cardiovascular, respiratory, and immune processes, which contribute to brain and body homeostasis. Furthermore, it is significant with respect to growth, as growth hormone (IGF-1) is produced during sleep [14,15,16]. Apart from the physiological and mental aspects affected by the problematic internet use, scientific evidence has highlighted its link with emotional aspects as well; research has shown that extensive internet use shares a bidirectional relationship with increased feelings of loneliness [17]. A study conducted in 2009 examined loneliness feelings of five different conditions; participants were assigned to different groups of face-to-face interaction, online chatting, watching a video, writing an essay and being completely inactive, and those who exhibited the higher levels of loneliness were those who were online chatting [18]. In addition, problematic internet use has been linked with low self-esteem; a study conducted in 1552 Chinese adolescents revealed that the higher the level of internet use, the lower the participants’ self-esteem was [19]. Several studies have examined the association between the time spent online on various devices and sleep. In the majority of these studies delayed sleep onset and bad quality of sleep were associated with increased time spent on internet [20,21]. A longitudinal study which was conducted in Taiwan and included 1253 children and adolescents, researchers revealed the relationship of internet use and sleep duration, where those who demonstrated an addictive behavior regarding internet had less sleep compared to regular users [22]. Another study which involved preadolescents and adolescents, and particularly 850 participants, exploring the effects of technology use, came to similar conclusions; problematic internet use significantly predicted decreased sleep duration [23]. Though there have been scientific efforts to systematically appraise the effect of media on adolescent life, the majority of these focused on the health outcomes on a physical or a mental state level, such as obesity or depression. Additionally, similar attempts have indeed focused on the effect of problematic internet use and sleep [24,25]. Alimoradi et al. conducted a systematic review and quantitative analysis on the effect of internet overuse on sleep and concluded in the fact that excessive internet users had 2.2-fold greater chance to demonstrate sleeping problems and almost half an hour less sleep compared to regular users. This study, although well and strictly designed, did not focus on adolescents [25]. Another systematic review conducted a few years earlier (2014) tried to explore the relationship between sleep and internet use and concluded in a negative relation of the two variables. Nevertheless, authors outlined that this relationship was weak, could be considered only as suggestive, and this finding was mostly due to the biases resulting from the design of the included studies. Furthermore, this study also included papers regardless of the participants’ age resulting in including mainly adult populations over 18 years of age [24]. Another systematic review focused on the same subject; yet among the included studies, there were papers examining the mediating effect of internet gaming addiction which is an established mental disorder [24]. In addition, other reviews focused on media in general, including television watching or video gaming [26]. Given the extended use of internet in adolescence, this study aimed to systematically review the evidence regarding the effects of problematic internet use on sleep during this important developmental stage.

### Aim of This Study

The primary objective of this review was to identify the possible effects of problematic internet use, without focusing on one type of medium and evaluated solely adolescent sleep. To the authors’ knowledge, there is no systematic review focusing on problematic internet use of this particular age group and its effect on sleep without the admixture of adolescents with young adults, and without taking into consideration the mediating role of specific mental or physical conditions. 

## 2. Methods

This systematic review investigated the effect of problematic internet use on adolescent sleep. The procedure and findings were reported according to the Preferred Reporting Items for Systematic Reviews and Meta-Analyses (PRISMA) Statement [27] so as to indentify papers that were relevant to the topic. Stages of research in detail included problem formulation, thorough search of the existing scientific research on the subject, data evaluation and finally data analysis and presentation. 

### 2.1. Eligibility Criteria

Inclusion criteria were the following: Original research studies of adolescent participants aged 10 to 19 years, as the adolescence life span is established by the World Health Organization [28] were eligible for inclusion. Studies had to be published within the last decade (2009–2020). In order for a study to be included in this review, it had to be of observational design so as to be homogeneous to facilitate the evaluation of their quality. Included studies had to investigate the effect of excessive internet use on sleep irrespective of the device used (i.e., mobile phone, computer, tablet) or the purpose of connection (i.e., leisure activity, social networking, academic purpose). Due to the lack of a scientifically “safe” time limit of internet use during adolescence [29], studies had to provide a distinct cut-off point for PIU, thereby using an established instrument for the assessment of this parameter. In order for a study to be eligible for inclusion, it had to be published in the English language and solely from peer-reviewed journals. 

Exclusion criteria were the following: Papers published in other languages apart from English and from not peer-reviewed journals were excluded. Studies that evaluated the adverse relationship, the effect of sleep on internet use, were also excluded. Studies that focused on population with a chronic mental or physical illness were not included, because of the known effects psychotropic or other systematic medication can have on sleep [30]. Other systematic reviews or meta-analyses were excluded. Relevant research protocols which did not provide sufficient results were not included either. 

### 2.2. Search Strategy

A comprehensive search of Pubmed and Scopus databases was performed from the 20 May 2020 until the 5 June 2020 to identify all articles that investigated the effect of problematic internet use on adolescent sleep published within the last decade (2009–2020). The process was conducted separately by two reviewers. Search terms used in each database and adopted accordingly if and when necessary were as shown in Table 1.

**Figure 1:**
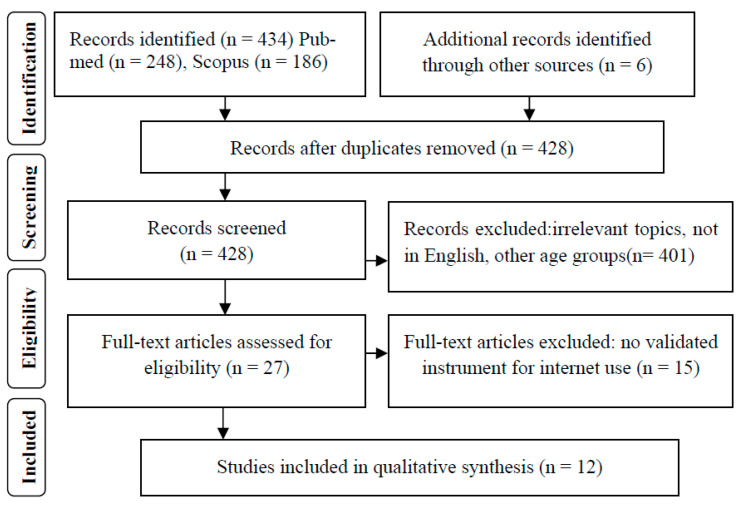
Flow chart of studies included in the review according to PRISMA guidelines.

The title, keywords and abstract of each study were screened for eligibility. The reviewing investigators independently searched introductions and reference lists of identified papers for additional studies relevant to the topic and then assessed all papers they resulted in according the inclusion/exclusion criteria. The search strategy was designed so as any discrepancies between the two reviewing investigators on whether a paper was eligible for inclusion to be resolved through discussion until a consensus was reached. However, no differences occurred during the final step of screening. Complete screening procedure is illustrated in Figure 1.

### 2.3. Data Extraction and Quality Evaluation

Data extraction was conducted by two reviewers. Data included country of origin, the sample size of each study, participants’ sex and age, the measurements used to evaluate internet use and sleep, the main outcomes of individual studies and any information required for the quality evaluation. The Appraisal Tool for Cross-Sectional Studies (AXIS) was used to assess studies’ quality [31]. AXIS consists of 20 items with each measuring a different aspect of a study’s quality. The aim of the tool is to aid systematic interpretation of an observational and, in particular, cross-sectional study and to assist decisions about the quality of the study being evaluated. Each question of the tool can be answered with “yes”, “no” or “I do not know”, yet it is not used to generate a total quality score, due to the well-known problems associated with such scores [32].

## 3. Results

### 3.1. Study Selection and Basic Characteristics

Initial search yielded 434 studies. After excluding irrelevant papers and those matching to the subject but not complying with the eligibility criteria, the final step of the screening process resulted in 12 observational studies. All of the included papers were of cross-sectional design. 

Included studies focused on sleep related behaviors and sleep problems that occur in adolescents due to PIU. Specifically, the papers retrieved primarily evaluated how problematic internet use affects the sleep quality and quantity, and some of them identified probable affecting factors mediating the relationship of the problematic internet use with sleep. The basic characteristics of individual studies are presented in Table 2. 

Included studies were published from 2009 until 2020. Eleven of them were held in Asian countries, and one was conducted in Europe. Ten of the studies aimed to assess the outcome of problematic internet use on adolescent sleep; for 2 of them the effect of problematic internet use on health-related behaviors in general was assessed, but sleep was one of the outcomes measured and evaluated separately. All studies used an established instrument for internet use assessment, providing a distinct cut off point for PIU. With respect to sleep assessment, 5 of them used inquiries on sleep duration and sleeping habits structured by each study’s researchers, while 7 of them used established, validated self-report questionnaires. Among the included papers, 18,987 adolescents were included in total, with the mean sample size being n = 1582.25, and the largest sample being n = 4750 and the smallest n = 180. Mean age of participants was 15.63 years, and the percentage of females across 11 of the studies was 50.6%, while in one of them solely females participated. Regarding the main outcomes of the problematic internet use-sleep relationship, 50% of the included studies reported a negatively affected sleep quality, and 50% of them reported shorter sleep duration. Among the studies 24% reported that the PIU-sleep relationship was mediated by the adolescent’s sex and the parents’ educational and social status, while 24% supported that the purpose of internet use was mediating the relationship.

### 3.2. Quality Evaluation

The assessment of the quality of individual studies was performed with the Appraisal Tool for Cross-Sectional Studies(AXIS) that addresses study reporting and overall quality [31]. The tool consists of 20 components, and the key domains addressed are study design, sample size justification, target population, sampling frame, sample selection, measurement validity and reliability, and methods. The overall quality of included studies could be characterized as highly justifying, with main issues arising with respect to sample size justification and discussion of limitations, both only for a few of the included studies. An additional issue emerged regarding the instruments used for sleep problems assessment, as 5 out of the 12 studies used inquiries structured by the researchers and not validated self-report or other instruments. Detailed results of the quality evaluation are presented in Table 3. The different methodological approach of included studies with respect to measurements makes the quantitative analysis not feasible. 

## 4. Discussion

As internet has become the main vehicle for entertainment, education and interaction with others, negative effects of the particular means of communication have gone under the microscope of clinicians and researchers. Studies up to date have highlighted the impact of problematic internet use on various aspects of the psychosocial, mental, and physical health of different age groups. The aim of this systematic review was to examine the body of evidence regarding the effect of problematic internet use on adolescent sleep. Among the results, the most important findings suggested that internet use affects sleep efficacy with various ways; the higher the use, the worse the sleep an adolescent receives is both on a quantitative and a qualitative level; the bright light emitted by devices seems to affect the sleep onset; excessive internet use seemed to cause insomniac symptoms. Furthermore, problematic internet use was associated with sleep medication intake. In addition, it appeared that certain sociodemographic adolescent and parental characteristics affected the level of internet use. The main findings of this study are discussed below.

### 4.1. Sleep Efficacy

Several of the included studies reported an influence of problematic internet use on sleep efficacy, which included sleep quantity and quality. With respect to sleep quantity, results regarding the relation between the extent of internet use and sleep showed that the higher the use, the more severe the effect on sleep duration was. One study found a weak, but significant positive correlation between sleep duration and problematic internet use [38]. Similarly, another reported that when comparing problematic users’ with regular users’ sleep duration, the latter had at least one more hour of sleep [33]. Canan et al. likewise found that decreased sleep duration was reported among severely problematic internet users compared to regular users [34]. This was the only one of the included papers supporting an implication of the problematic internet use with sleep duration; those who scored higher in the internet use measure were those who reported shortened total sleep time. A finding worth noting of this study was the fact that apart from problematic users, moderate users were also likely to demonstrate prolonged sleep latency, and thereby less sleep. This finding came in line with another study on Taiwanese college students, were internet use was strongly correlated with later bedtime [45]. A study conducted in Turkey discovered that PIU caused later bedtime and longer sleep onset latency, which resulted in fewer hours of sleep [35]. Similar findings were reported by Choi et al. who also highlighted the decreased duration of sleep for problematic compared to regular internet users and the daytime sleepiness in which sleep deprivation resulted in [36].The finding of shorter sleep due to problematic internet use came in line with what was reported in the study of Kawabe et al.; total sleep time was significantly correlated with increased internet use, and more particularly, those who scored higher in the internet use scale had shorter nocturnal sleep when compared to regular users [37]. Interestingly, this study highlighted the need which problematic internet users demonstrated for a later sleep offset during weekends. The tendency for sleep regain following deprivation means that sleep is more than body’s inactivity [46]. The results of the mentioned findings came in accordance with a study on adolescents where those who demonstrated an addictive behavior regarding internet had less sleep compared to regular users [22]. Inadequate sleep due to problematic internet use may be abetted by the chronotype’s shift; it has been observed that during the second decade of life sleep/wake times shift later and adolescents seem to sleep later as they get older [47]. A further contributing factor to sleep displacement could be the exposure to bright light before sleep. Exposure to the bright light of devices can cause a shift to later hours due to melatonin suppression [48]. Melatonin is a hormone that regulates circadian rhythm and synchronizes the sleep-wake cycle. By doing so, it assists the transition to sleep and promotes consistent rest [49]. Adequate sleep during adolescence is of great importance; according to Wolfson and Carskadon’s study, adolescents need at least 9 h of sleep, with those sleeping less demonstrating higher risk of obesity and learning difficulties [50]. Insufficient adolescent sleep could be of great concern, as literature has shown that behaviorally decreased sleep and weekend oversleep may lead to suicidal tendencies independently of depression existence [51]. Qualitative sleep is of great importance for children and adolescents as, apart from its biological role, it is highly related to learning ability, memory and school performance [52,53]. High-quality sleep is characterized by no nighttime disturbances such as sleep interruption, daytime dysfunction and issues such as bruxism. The majority of the included studies revealed that problematic internet use and sleep quality seem to have a dose–response relationship. Researchers showed that adolescent interactive online activities such as social networking with their peers along with higher internet use scores resulted in bad quality of sleep [39]. One study showed that excessive internet use significantly predicted sleep disturbances [40] and another study similarly found irregular sleeping patterns and increased sleep disturbance for problematic users [41]. The impact of problematic internet use on sleep quality was found to apply to regular users too; 25% of the average users had bad quality of sleep as well [33]. Two studies showed that the higher the score on internet use, the worse the quality of participants’ sleep was [38,42]. The negative relation between problematic internet use and sleep was also reported by a study; sleep quality was poorer for those who exhibited higher internet use [43]. Specific problems negatively affecting sleep quality were reported in one of the studies, with problematic internet users experiencing symptoms such as snoring, apnea and nightmares in a greater degree than regular users. Sleep quality is of great importance, as it affects the sensitive, developmental stage of adolescence in various ways [43]. Literature has shown that the effect may be physiological or psychological [54] and can lead to obesity [55] and anxiety [56]. Apart from these health-related risks, bad sleep quality has been related to low academic performance [57] and substance use [58].

### 4.2. Insomnia Symptoms

Though insomnia is the most prevalent sleep disorder observed during adolescence [59], few of the included studies investigated the correlation of this sleep parameter with the internet use. Excessive internet use may cause problematic sleeping conditions such as insomnia or insomnia symptoms, as it was shown in two of the included studies. They revealed that the time spent online was associated with insomnia. Adolescents with more than five hours of daily internet use demonstrated insomniac symptoms, yet this relation was intervened by the effect of the school workload of the participants [39]. Accordingly, a different study discovered that the higher the internet use, the more often insomnia symptoms occurred. In this study, problematic users reported more frequently insomniac symptoms than average users and average users more frequently than non-users, something that subsequently led to excessive daytime sleepiness [36]. Both studies’ results came in line with the findings of a research on the same subject but in an adult age group, which demonstrated significant correlation among insomnia and PIU. Adolescents who suffer from sleep deprivation are at high risk of developing several mental issues such as anxiety and depression [60] and, in more advanced complications, suicidal ideation [61].

### 4.3. Use of Sleep Medication

Though only reported by two of the included studies, use of sleep medication, especially at such an early age, is a parameter worth to discuss. Only these, of all the studies, reported that problematic internet use was associated with hypnotic medication intake [38]. The same observation was made in the study of Lin et al., who reported that internet overuse was positively correlated with the use of sleeping medication [42]. Problematic internet use can cause emotions such as anxiety [62] or interpersonal and emotional difficulties [63], and these difficulties may create a vicious circle between them and sleeping problems. A plausible explanation could be the fact that the intake of this type of medication may function as a soothing mechanism for negative emotions.

### 4.4. The Role of Sex, Parental Characteristics, Purpose and Time of Use

An interesting parameter that seems to have a role in the relationship of problematic internet use with sleep is adolescent’s sex. While one of the included studies found no significant difference between males and females [36], another found that problematic internet use was higher for male participants [33]. On the contrary, three other studies showed that the prevalence of problematic internet use was higher for females [38,43,44]. Based upon the contradictory findings, additional research on the role of the adolescent’s sex could be enlightening; mood and sleep share common genetic ground, have a bidirectional relationship and females are more likely to experience mood disorders [64], thereby sleep may affected. Additionally, the hormonal fluctuation females undergo during adolescence may significantly affect their sleep/wake pattern [65], and problematic internet use may not be the real cause of sleep abnormalities.

Specific parental characteristics appeared to be potential affecting factors in the relationship of problematic internet use with adolescent sleep. The study of Tan et al. showed that the level of parental education had an effect on the use of internet by adolescents. For male adolescents, paternal high educational level was a predictive factor for internet overuse [40]. In the same study, the type of family appeared to have its role in the problematic internet use-sleep relationship as well; researchers reported that coming from a non-intact family was a predictive factor for internet overuse and sleep problems [40]. In one of the studies, it was found that a combination of low parental educational and financial level was related to high scores in internet use and negative results in sleep measurements [40]. Literature has shown that parental educational level has an important role in the behavioral outcomes of adolescents [66]. This could be explained by the stimulus for other, more beneficial activities or by more efficient ways of parental supervision one with higher education can offer or by the type of relationship they can develop with their child. In fact, there have been studies which showed the effect of the parenting style on the internet use of adolescents; high engaging and supportive parenting behaviors were negatively linked to adolescent internet addiction, whereas adolescents who perceived their parents as hostile and rejecting were likely to develop problematic internet behaviors [67].

In terms of the purpose of internet use, one of the studies revealed that problematic internet use negatively affected sleep when the purpose of use was associated with leisure activities, such as chatting and online gaming but not when the use occurred due to academic reasons, such as e-mail checking or studying [33]. Researchers found that sleep deprivation was related to the purpose of use, as adequate sleep was negatively correlated with leisure internet time, as it appeared to eliminate stress and enhance self-esteem and autonomy [41]. This could be possibly explained by the nature of the use. Internet use requires either a passive or active engagement. Active engagement such as online chatting, in contrast to passive net browsing, can be linked to psychological arousal and thereby sleep delay. The delay of sleep initiation may be explained by the prolonged thought process and cognition alertness caused by the active engagement.

### 4.5. Limitations of This Study

The main strengths of this study are the large sample sizes justified by the study’s design, and the criterion of widely used instruments regarding PIU, in order to avoid any subjective bias. This review, however, bears certain limitations. Self-report questionnaires used in all studies are not equivalent to clinical evaluation, and thereby, results could have been affected by report biases and objective conclusions cannot be drawn with certainty. Additionally, observational studies cannot prove a causal relationship between problematic internet use and sleep problems, and other factors such as some of those mentioned above could have a stronger role than internet use in disturbed adolescent sleep. Furthermore, a quantitative analysis of the results was not feasible. Due to the heterogeneity of the studies’ instruments, only three studies used the same instruments with respect to internet use and sleep indexes. Without a sufficient number of studies, a problem estimating the between-studies variance would emerge, which would have important implications for many aspects of the analysis.

### 4.6. Clinical Importance and Recommendations for Future Research

The findings of this review highlighted the possible effect excessive internet use can have on the sleep of adolescents, in such a crucial developmental stage. Clinicians and educators can address this issue to parents and caregivers and discuss the importance of health sleeping patterns and routines, how important avoiding internet use before bedtime is and the catastrophic consequences of exposure to bright light in the evening can have. In addition, they should remind them the significance of paying attention to behavioral changes with respect to internet use.

Future research on the field should take into consideration the various sociodemographic characteristics affecting the problematic internet use–sleep interaction, so the cause–effect relationship can be defined more precisely. Studies of longitudinal design which are population based and use valid and widely used instruments should be conducted. This would assist the effort to completely comprehend the effect of internet use on adolescent life. Furthermore, experimental studies of well-controlled design would help to shed light on the alterations of the circadian rhythm and other related to sleep mechanisms.

## 5. Conclusions

Teenagers seem to spend increasing time on online activities using several technological devices (smart phones, tablets, laptops and computers) for various educational and recreational purposes on a daily basis. This everyday habit can become a health risk factor, since it can take the form of a behavioral exaggeration and affect different physiological and mental aspects of health. This can be crucial for an individual’s overall health status, as adolescent health risk behaviors are known to be carried into adulthood. One of the parameters affected is sleep as it gets shorter, becomes irregular, and sleep onset and offset are highly influenced by problematic internet use. This may occur either due to sleep displacement and circadian rhythm shifts, increased psychological and cognitive arousal or due to melatonin suppression that occurs to exposure to bright light before sleep. The present systematic review showed that there is a wide field offered for research and prevention on the subject. The most current statistics show that globally more than 3.5 billion people, among them mostly the young, access internet via social media platforms. As far as prevention is concerned, adolescents as well as parents can be educated about the need of moderate use of internet and the adverse effects of problematic internet use on adolescent sleep. Sleep quality and efficiency are important components of living a healthy life. Adolescents could benefit from educational programs or from advice from physicians well informed on the subject, which will reinforce the responsibility towards their health status. Programs involving parents should be informative about the need for a supervised internet use with a pre-specified timeframe. With regards to research, the acknowledgement of specific psychological or social traits of users which can lead some but not all to problematic internet use, allows to focus on supporting individuals who are at higher risk.

## Figures and Tables

**Table 1:** Search terms used for papers’ identification.

Terms for Internet Use	And	Terms for Sleep
problematic internet use		sleep
Or		Or
internet addiction		sleep deprivation
Or		Or
internet use		sleep problems

**Table 2:** Characteristics of included studies.

Study Ref. #,Year, Origin	Participants Sample, Mean Age, Sex (Females)	Measurements on Internet Use	Measurements on Sleep	Main Results
[33] 2014, Switzerland	n = 3067, 14.23 years, 50.3%	Young’s IAT	Structured inquiry on sleep	PI users had at least 1 h less sleep that those who weren’t. PIU was higher in females. 80% of problematic internet users used it for leisure.
[34] 2013, Turkey	n = 1024, 16.04 years, 52.5%	Young’s IAT	Structured inquiry on sleep	PIU was associated with decreased sleep but only when used for leisure. PIU was higher for males.
[35] 2014, Turkey	n = 1212, 16 years, 52.6%	Young’s IAT	Semi-structured inquiry on sleep	PI users had higher frequencies of sleep problems (later bedtime, delayed sleep onset, night awakenings)
[36] 2009, South Korea	n = 2336, 16.7 years, 42.5%	Young’s IAT	ESS, structured inquiry on sleep problems	PI users slept significantly less than regular users. Increased sleeping problems among severe users (insomnia symptoms, snoring, teeth grinding, nightmares). Insomnia symptoms were higher in older adolescents
[37] 2019, Japan	n = 853, 13.6 years, 50.17%	Young’s IAT	CASC	Shorter sleep duration, later sleep onset (weekdays and weekends) and later sleep offset (weekends) for problematic users.
[38] 2020, Turkey	n = 1487, 16.16 years, 39.4%	Young’s IAT	PSQI	PIU negatively affects sleep quality. Weak but positive association with sleep duration–efficiency–latency and use of sleep medication. Using internet before bedtime was associated with higher PIU.
[39] 2018, Indonesia	n = 180, 17.0 years, 65%	SMSAQ	Insomnia questionnaire	Duration of use was significantly correlated with insomnia symptoms. High tolerance was the main reason of problematic use.
[40] 2016, China	n = 1661, 14.53 years, 48.2%	Young’s IAT	PSQI	PIU was associated with sleep disturbances. Higher level of father’s education was associated with PIU, but only for males.
[41] 2010, Korea	n = 853, 14.0 years, 55.2%	Korean Internet Addiction Scale	Structured inquiry on sleep	PIU was associated with irregular bedtime patterns and sleep disturbance. Low parental income and educational level associated with PIU.
[42] 2019, Taiwan	n = 503, 17.05 years, 100%	Young’s IAT	PSQI	PIU was associated with low sleep quality-latency-duration and use of sleep medication.
[43] 2016, Turkey	n = 1061, 16.2 years, 50.0%	IAS	PSQI	PIU was associated with poor sleep quality and lack of parental supervision.
[44] 2018, China	n = 4750, 16.0 years, 50.8%	Young’s IAT	PSQI	PIU associated with elevated risk of sleep disturbance. Sleep disturbance was higher in females, older adolescents were at greater risk for sleep disturbance.

ABBREVIATIONS: IAT = Internet Addiction Test, PSQI = Pittsburg’s Sleep Quality Index, ESS= Epworth Sleepiness Scale, CASC = Child and Adolescent Sleep Checklist. Notes: **#**: Symbol refers to the reference number in the references sections

**Table 3:** Quality assessment of studies using the AXIS tool.

	Study **											
Question	38	43	42	35	33	44	36	37	39	33	40	41
Were the aims/objectives of the study clear?	Y	Y	Y	Y	Y	Y	Y	Y	Y	Y	Y	Y
Was the study design appropriate for the stated aim(s)?	Y	Y	Y	Y	Y	Y	Y	Y	Y	Y	Y	Y
Was the sample size justified?	Y	Y	Y	Y	Y	Y	Y	Y	N	N	N	Y
Was the target/reference population clearly defined? (Is it clear who the research was about?)	Y	Y	Y	Y	Y	Y	Y	Y	Y	Y	Y	Y
Was the sample frame taken from an appropriate population base so that it closely represented the target/reference population under investigation?	Y	Y	Y	Y	Y	Y	Y	Y	Y	Y	Y	Y
Was the selection process likely to select subjects/participants that were representative of the target/reference population under investigation?	Y	Y	Y	Y	Y	Y	Y	Y	Y	Y	Y	Y
Were measures undertaken to address and categorize non-responders?	Y	Y	Y	N	N	N	Y	Y	N	Y	N	Y
Were the risk factor and outcome variables measured appropriate to the aims of the study?	Y	Y	Y	Y	Y	Y	Y	Y	Y	Y	Y	Y
Were the risk factor and outcome variables measured correctly using instruments/measurements that had been trialled, piloted or published previously?	Y	Y	Y	N	N	Y	Y	Y	Y	N	Y	Y
Is it clear what was used to determined statistical significance and/or precision estimates? (e.g., *p*-values, confidence intervals)	Y	Y	Y	Y	Y	Y	Y	Y	Y	Y	Y	Y
Were the methods (including statistical methods) sufficiently described to enable them to be repeated?	Y	Y	Y	Y	Y	Y	Y	Y	N	Y	Y	Y
Were the basic data adequately described?	Y	Y	Y	Y	Y	Y	Y	Y	Y	Y	Y	Y
Does the response rate raise concerns about non-response bias?	N *	N *	N *	N *	N *	N *	N *	N *	DK	N *	N *	N *
If appropriate, was information about non-responders described?	Y	Y	Y	N	Y	N	N	Y	N	Y	Y	Y
Were the results internally consistent?	Y	Y	Y	Y	Y	Y	Y	Y	Y	Y	Y	Y
Were the results presented for all the analyses described in the methods?	Y	Y	Y	Y	Y	Y	Y	Y	Y	Y	Y	Y
Were the authors’ discussions and conclusions justified by the results?	Y	Y	Y	Y	Y	Y	Y	Y	Y	Y	Y	Y
Were the limitations of the study discussed?	Y	Y	Y	Y	Y	Y	N	Y	N	Y	Y	N
Were there any funding sources or conflicts of interest that may affect the authors’ interpretation of the results?	DK	N *	DK	N *	N *	N *	DK	N *	DK	DK	N *	N *
Was ethical approval or consent of participants attained?	Y	Y	Y	Y	DK	Y	DK	Y	Y	Y	Y	Y

Notes: Y= yes, N = no, DK = do not know, ** = Numbers refer to the number assigned to each study in References, * = a negative answer for questions “Does the response rate raise concerns about non-response bias?” and “were there any funding sources of conflicts of interest that may affect the authors’ interpretation of the results?” are reversed questions, and a negative answer is considered positive.

## Data Availability

No new data were created or analyzed in this study. Data sharing is not applicable to this article.
